# Evaluación de las características operativas de la versión 2.2018 del instrumento de evaluación del malestar emocional de la *National Comprehensive Cancer Network* en pacientes atendidos en el Instituto Nacional de Cancerología, Bogotá

**DOI:** 10.7705/biomedica.6131

**Published:** 2021-12-15

**Authors:** Sofía Elizabeth Muñoz, Ricardo Sánchez, Ligia Elena del Toro

**Affiliations:** 1 Grupo Área de Investigaciones, Instituto Nacional de Cancerología - E.S.E., Bogotá, D.C., Colombia Grupo Área de Investigaciones Instituto Nacional de Cancerología - E.S.E. Bogotá D.C. Colombia; 2 Facultad de Medicina, Universidad Nacional de Colombia, Bogotá, D.C., Colombia Universidad Nacional de Colombia Facultad de Medicina Universidad Nacional de Colombia Bogotá D.C. Colombia; 3 Grupo de Salud Mental, Instituto Nacional de Cancerología - E.S.E., Bogotá, D.C., Colombia Grupo de Salud Mental Instituto Nacional de Cancerología - E.S.E. Bogotá D.C. Colombia

**Keywords:** estudios de validación, comparación transcultural, escala del estado mental, distrés psicológico, neoplasia, sensibilidad y especificidad, Validation studies, cross-cultural comparison, mental status schedule, psychological distress, neoplasm, sensitivity and specificity

## Abstract

**Introducción.:**

Los pacientes con cáncer presentan niveles significativos de malestar emocional. La *National Comprehensive Cancer Network* (NCCN) desarrolló un instrumento (*Distress Management*) para evaluarlo de forma rápida en pacientes oncológicos. Para su utilización en Colombia, se hizo la adaptación transcultural y se validó.

**Objetivo.:**

Determinar las características operativas del instrumento de malestar emocional, versión 2.2018, en pacientes atendidos en el Instituto Nacional de Cancerología.

**Materiales y métodos.:**

Previa autorización de la NCCN, se procedió a la traducción, adaptación transcultural y evaluación de las características operativas del instrumento. Se incluyeron 343 pacientes con diagnóstico de cáncer atendidos en el Instituto Nacional de Cancerología, quienes diligenciaron el instrumento adaptado transculturalmente. Se efectuó un estudio de prueba diagnóstica como patrón de referencia mediante una entrevista semiestructurada.

**Resultados.:**

Los pacientes tenían una edad promedio de 49,7 años (DE=15) y la mayoría (67 %) eran mujeres. El instrumento tuvo un área bajo la curva ROC de 0,81 (IC_95%_ 0,77- 0,86); el punto de corte óptimo fue de 3,5, el cual se aproximó a 4; la sensibilidad fue de 0,81 (IC_95%_ 0,76-0,85) y la especificidad de 0,69 (IC_95%_ 0,64-0,74). El porcentaje de acuerdo entre el resultado de la entrevista y el instrumento fue de 73 % (kappa=0,64; p<0,001).

**Conclusiones.:**

El instrumento de malestar emocional permitió detectar el malestar emocional moderado a grave que requiere intervención y manejo. Este instrumento fue adaptado y validado en pacientes con cáncer en Colombia, conservándose el punto de corte en ≥4 como en la versión original.

## Abstract (en)

**Introduction:**

Cancer patients have significant levels of emotional distress. The National Comprehensive Cancer Network (NCCN) developed the distress management tool to quickly assess significant distress in oncological patients who require intervention. For its use in Colombia, we made its cross-cultural adaptation and validation.

**Objective:**

To determine the operative characteristics of the distress management tool, version 2.2018, in patients seen at the *Instituto Nacional de Cancerología* (INC) in Colombia.

**Materials and methods:**

Counting with the authorization from the NCCN, we translated, made the cross-cultural adaptation, and evaluated the operational characteristics of the tool. We included 343 cancer patients seen at the INC, who filled out the cross-culturally adapted instrument. A diagnostic test study was carried out with a semi-structured interview as a reference.

**Results:**

The patients had an average age of 49.7 years (SD=15) and the majority were women (67%). The instrument had an area under the ROC curve of 0.81 (95% CI: 0.77 - 0.86); its optimal cut-off point was 3.5 approached to 4 when using integers on the scale; its sensitivity was 0.81 (95% CI: 0.76 - 0.85), and its specificity, 0.69 (95% CI: 0.64 - 0.74). The agreement percentage between the result of the interview and the instrument was 73% (kappa = 0.64; p< 0.001).

**Conclusions:**

The distress management tool allowed for the detection of moderate to severe distress requiring intervention and management. This instrument was adapted and validated in cancer patients in Colombia keeping the cutoff point at ≥ 4 as in the original version.

Con el aumento de la incidencia de cáncer a nivel mundial en los últimos años, ha crecido el interés por el reconocimiento del malestar emocional (*distress*) en pacientes oncológicos. Teniendo en cuenta que el diagnóstico de cáncer y su tratamiento generan un impacto psicológico en ellos, estas manifestaciones clínicas y emocionales pueden ser adaptativas, asociadas a su proceso de enfermedad, o llegar a ser un trastorno psiquiátrico grave que requiere manejo, lo cual puede depender de características sociodemográficas, del diagnóstico oncológico, y del estadio de la enfermedad y del tratamiento, entre otros ^1^^-^^3^.

En algunos estudios se ha determinado que entre el 20 y el 50 % de los pacientes con cáncer presenta alteraciones psicológicas, incluido el malestar emocional, siendo los trastornos de la adaptación, los de ansiedad y la depresión los más frecuentes ^3^^-^^7^. A pesar de la alta prevalencia de malestar emocional, a menudo este no es detectado ^8^; de ahí la importancia de detectarlo para brindar soporte emocional temprano y atención integral al paciente, ya que las necesidades psicosociales no resueltas producen efectos negativos en el tratamiento oncológico y el proceso de recuperación, afectando el cumplimiento del tratamiento, aumentando el sufrimiento del paciente y comprometiendo su calidad de vida y la de sus cuidadores ^8^^-^^12^.

Las recomendaciones de la *National Comprehesive Cancer Network*, el *Institute of Medicine*, la *Canadian Association of Psychosocial Oncology* y la *American College of Surgeons Commission on Cancer* en cuanto a la atención psicosocial de pacientes con diagnósticos oncológicos, señalan la necesidad de establecer protocolos para estandarizar la evaluación de los aspectos psicosociales de la atención del paciente con cáncer. Dicha evaluación debe hacer parte integral de la rutina de la atención oncológica ^4^^,^^13^^-^^18^. Para evitar la estigmatización de los pacientes de cáncer con problemas psicológicos, la NCCN propuso usar el término *distress,* que en español se conoce como malestar emocional, para referirse a las dificultades a nivel psicológico, social o espiritual que interfieren con la capacidad de enfrentar su proceso de enfermedad, las cuales deben ser reconocidas y tratadas en todas sus etapas ^4^.

En Colombia, la atención de los pacientes oncológicos presenta retos para los profesionales de salud, pues el número de enfermos atendidos diariamente es alto y el tiempo es limitado, lo que provoca que el profesional se centre en la enfermedad y no le sea fácil reconocer el malestar emocional, con la consecuente ausencia de estrategias para el diagnóstico y manejo de tales problemas ^3^. Es patente, entonces, la necesidad de contar con herramientas rápidas y eficaces de tamización para detectar estos problemas. Son muchas las herramientas que se han desarrollado para detectar síntomas psicológicos y psiquiátricos en condiciones específicas como la depresión o la ansiedad, por ejemplo, el inventario de depresión de Beck, la escala de Hamilton, la escala de ansiedad y depresión hospitalaria (*Hospital Anxiety and Depression Scale*, HADS), las escalas de calidad de vida ^16^^-^^19^ y el listado de detección de problemas psicosociales (*Severity Indices of Personality Problems*, SIPP) ^20^, entre otros. Sin embargo, las herramientas cortas exploran solo una dimensión del malestar y tienen poca especificidad a la hora de detectarlo, en tanto que las herramientas más extensas pueden abarcar todas las dimensiones, pero son más difíciles de utilizar en el ámbito clínico por su duración y complejidad ^21^.

En respuesta a dichos problemas, Roth, *et al.*, desarrollaron en 1998 un instrumento corto diligenciado por el propio paciente y similar a la escala visual análoga en la que 0 corresponde a la ausencia total de malestar y 10 a un malestar extremo. Esta herramienta, llamada “termómetro de malestar emocional” (*Distress Thermometer*), fue utilizada por primera vez para evaluar de manera rápida el malestar emocional significativo en pacientes con cáncer de próstata que asistían a consulta ^22^. Posteriormente, la NCCN agregó una lista de problemas en cinco dominios (problemas prácticos, familiares, emocionales, espirituales y físicos), con el fin de recabar la información mínima que permitiera hacer una remisión más específica a los servicios especializados de psicología, psiquiatría, trabajo social y servicio religioso, la cual se usa en todos los pacientes con cáncer en diferentes estadios y recibió el nombre de *distress management*^4^.

La NCCN sugiere que una puntuación igual o mayor de 4 en la escala indica un malestar emocional clínicamente significativo que requiere atención y manejo ^23^. Este instrumento ha sido traducido y validado en diversos países, manteniendo características operativas similares, con un punto de corte que oscila entre 3 y 5, y un área bajo la curva entre 0,7 y 0,88, según los países y culturas ^4^^,^^23^^,^^24^. La mayoría de los estudios han reportado un resultado de 4, como en la versión original, lo que permite usar el instrumento en todas las enfermedades oncológicas en sus diferentes estadios y en diferentes tiempos, ya que indaga sobre el malestar presente durante la semana anterior, incluido el día del diligenciamiento del instrumento.

Aunque existe una versión del 2013 en español en la NCCN ^25^, se consideró que el estilo de redacción y el vocabulario dificultaban su comprensión en la población colombiana, por lo que se sometió la versión 2 del 2018 del instrumento a un proceso de adaptación transcultural. Dado que no se conocían las propiedades del instrumento para clasificar correctamente a los pacientes con malestar emocional que requieren intervención, el objetivo del presente estudio fue determinar las características operativas de dicha versión en pacientes con diagnóstico de cáncer atendidos en el Instituto Nacional de Cancerología - E.S.E. en Bogotá utilizando la versión ya traducida y adaptada transculturalmente ^26^. Se revisó la última versión disponible de la NCCN del 2020 y no se encontraron cambios con respecto a la que se tradujo y validó (versión 2.2018).

Este estudio contó con la aprobación del comité de ética del Instituto Nacional de Cancerología y con la autorización de los autores de la NCCN para usar el instrumento *Distress Management* (2018).

## Materiales y métodos

### Diseño

Se hizo un estudio de evaluación de las características operativas de un instrumento de medición.

### Población

Se trabajó con pacientes con diagnóstico de cáncer atendidos en los servicios de hospitalización y consulta externa en el Instituto Nacional de Cancerología. Se incluyeron hombres y mujeres mayores de 18 años, hispanohablantes, que supieran leer y escribir, y aceptaran participar en el estudio firmando el consentimiento informado.

### Instrumento

El instrumento *Distress Management* tiene dos partes: la primera parte consta de las instrucciones para su diligenciamiento y el termómetro de malestar emocional en el que el paciente debe encerrar en un círculo el número de 0 a 10 que describa cuánto malestar ha experimentado en la semana anterior, incluido el día de la aplicación del instrumento, siendo 0 ningún malestar y 10 el malestar extremo:

En la segunda parte se le pide al paciente que indique si algún ítem de la lista ha sido un problema para él en la semana anterior, incluido el día de trámite del instrumento, marcando Sí o No en cada opción de la lista de problemas agrupados en cinco dominios. Además, hay una pregunta final abierta para explorar otros problemas diferentes a los enunciados. El instrumento consta de un total de 41 ítems, incluida la pregunta abierta final (cuadro 1).

**Cuadro 1:** Versión colombiana del instrumento Distress Management, versión 2.2018

Problemas prácticos	Sí	No
Cuidado del hogar		
Cuidado de los niños		
Seguros o finanzas		
Transporte		
Trabajo o estudio		
Decisiones acerca de tratamientos médicos		
**Problemas familiares**	**Sí**	**No**
Interacción con los niños		
Interacción con la pareja		
Capacidad para tener niños		
Problemas de salud en la familia		
**Problemas emocionales**	**Sí**	**No**
Depresión		
Miedos		
Nerviosismo		
Tristeza		
Preocupación		
Pérdida de interés en actividades habituales		
**Preocupación a nivel espiritual o religioso**	**Sí**	**No**
**Problemas físicos**	**Sí**	**No**
Apariencia		
Bañarse o vestirse		
Respiración		
Cambios en la orina		
Estreñimiento		
Diarrea		
Alimentación		
Fatiga o cansancio		
Sensación de estar hinchado		
Fiebre		
Capacidad para moverse		
Indigestión		
Memoria o concentración		
Úlceras en la boca		
Náusea		
Nariz seca o congestionada		
Dolor		
Vida sexual		
Piel seca o picazón		
Sueño		
Uso de sustancias psicoactivas		
Hormigueo en manos o pies		
**Otros problemas:**	**Sí**	**No**
¿Cuales?		

A continuación, se reproducen las instrucciones tal como el paciente las lee:

1. “Instrucciones: Encierre en un círculo el número (del 0 al 10) que describa cuánto malestar ha experimentado usted en la semana pasada, incluyendo el día de hoy.
2. Lista de problemas: Instrucciones: Por favor indique si cualquiera de los siguientes ha sido un problema para usted en la semana pasada incluyendo el día de hoy, asegúrese de marcar Sí o No en cada opción”.

### Entrevista semiestructurada

Como ya se mencionó, esta se consideró como el estándar de referencia para fines del estudio de las características operativas del instrumento y estuvo a cargo de dos profesionales de salud mental, quienes evaluaron los requisitos de apoyo en cada uno de los dominios del instrumento.

### Procedimiento

Entre agosto y noviembre del 2018, se seleccionaron 343 pacientes mediante muestreo no probabilístico y secuencial conforme cumplían los criterios de elegibilidad. Cada paciente diligenció la versión en español del *Distress Management*, v 2.2018, adaptada transculturalmente para su uso en Colombia. El proceso inicial de traducción y adaptación fue publicado previamente ^26^, realizando posteriormente la evaluación de las características operativas del instrumento. Antes del diligenciamiento del instrumento, el equipo investigador explicó las dos partes y suministró información detallada sobre cómo debían diligenciarse. Además, en otro momento se le hizo a cada paciente una entrevista semiestructurada a cargo de un médico y un psicólogo, quienes determinaron si el paciente presentaba una situación de malestar emocional que requiriera intervención; estos dos profesionales eran expertos en salud mental con una experiencia de varios años en la evaluación de aspectos emocionales en pacientes con cáncer. La secuencia de la aplicación del instrumento y la entrevista se asignó de manera aleatoria; la entrevista semiestructurada estaba cegada con respecto al resultado del instrumento y se la consideró como el estándar de referencia para efectos del análisis de las características operativas.

En la fase de análisis, se evaluó la validez del instrumento mediante el estudio de la curva de características operativas del receptor (ROC) estimada a partir de los valores de sensibilidad y especificidad con el punto de corte estimado, y calculando la concordancia entre el instrumento y la entrevista semiestructurada.

El estudio fue aprobado por el comité de ética del Instituto Nacional de Cancerología el 6 de junio de 2019.

### Análisis estadístico

El análisis de los resultados se hizo con el programa estadístico Stata, versión 13. Fue un análisis descriptivo mediante el cálculo de medidas de tendencia central (medias y medianas) y de dispersión para las variables cuantitativas según los parámetros de normalidad (test de Shapiro Wilk); en tanto que, para las variables categóricas, se empleó un análisis de frecuencias absolutas y relativas. El punto óptimo de clasificación se estimó empleando el método propuesto por Perkins, *et al.*^27^, mediante una curva ROC. Se calcularon, además, la sensibilidad, la especificidad y los valores predictivos de la prueba, así como sus intervalos de confianza del 95 %. Para evaluar la concordancia entre la entrevista y el instrumento, se calcularon los coeficientes kappa ^28^.

## Resultados

La muestra incluyó a 343 pacientes que respondieron todos los ítems del instrumento en un tiempo promedio de cinco minutos. El 75 % de los participantes se seleccionó en los servicios de consulta externa y, el restante, entre los pacientes hospitalizados. Posteriormente, se hizo la entrevista semiestructurada, la cual tuvo una duración promedio de 30 minutos e incluyó la medición de las variables sociodemográficas. Los pacientes tenían un promedio de edad de 49,7 años (DE=15), la mayoría (67 %) eran mujeres, el 71% de ellos no tenía ocupación, el 59 % tenía pareja, el 38 % había terminado el bachillerato y el 34 % solo la primaria, el 59 % era de Bogotá y, de este porcentaje, el 90 % provenía del área urbana. En cuanto al nivel socioeconómico, el 35 % pertenecía a un estrato bajo (2) y el 65 % pertenecía al régimen subsidiado de salud; el 71 % era católico. Los cánceres más frecuentes fueron el de mama (21 %), el de útero (13 %), la leucemia linfoide (9,3 %), el linfoma no Hodgkin (9 %), el cáncer de colon y recto (8 %) y el de estómago (7 %). La mediana del tiempo transcurrido entre el diagnóstico y la aplicación del instrumento fue de 254 días (rango intercuartílico=597), el 34 % de los pacientes se encontraba en estadio III de la enfermedad y el 83 % recibía tratamiento con quimioterapia (cuadro 2).

**Cuadro 2:** Características de los pacientes

Variables	Frecuencia (n=343)	Porcentaje (%)
Edad*	49,7	15
Sexo		
Mujer	230	67
Hombre	113	33
Ocupación		
Sin ocupación	244	71
Con ocupación	99	29
Estado civil		
No tiene pareja	142	41
Sí tiene pareja	201	59
Nivel de educación		
Bachillerato	132	38,5
Universidad	115	33,5
Primaria	47	14
Maestría	28	8
Técnico	13	3,8
Especialización	7	2
Tecnólogo	1	0,2
Municipio procedencia		
Bogotá	202	59
Ibagué	12	3,5
Villavicencio	8	2,3
Yopal	7	2
San José del Guaviare	5	1,5
Soacha	5	1,5
Sogamoso	5	1,5
Características del municipio		
Urbano	310	90
Rural	33	10
Estrato socioeconómico		
2	121	35
3	99	29
1	96	28
4	20	6
5	6	1,8
6	1	0,2
Religión		
Católico	242	71
Otra	77	22
Sin religión	21	6
Evangélico	3	1
Aseguramiento		
Régimen subsidiado	224	65
Régimen contributivo	110	32
Régimen especial	9	3
Localización de cáncer		
Mama	71	21
Útero	46	13
Leucemia linfoide	32	9.3
Linfoma no Hodgkin	31	9
Colon y recto	29	8
Estomago	24	7
Leucemia mieloide	14	4
Próstata	12	3,5
Tiempo de evolución**	254	597
Estadio del cáncer		
III	117	34
0	90	26
IV	56	16
II	50	15
I	30	9
Tratamiento		
Quimioterapia	286	83
En espera	22	6
Otro	18	5
Cirugía	10	3
En recaída	5	1,5
Radioterapia	2	0,5
Sitio del INC		
Consulta externa	258	75,2
Hospitalización	85	24,8

*Promedio y desviación estándar (DE)

** Mediana y rango intercuartílico

INC: Instituto Nacional de Cancerología

**Figura 1:**
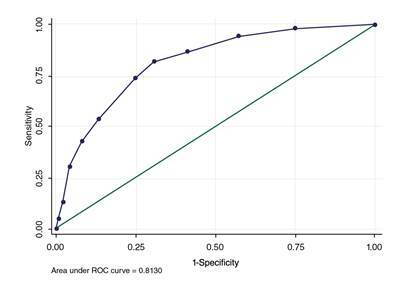
Curva ROC según la sensibilidad y especificidad de cada punto de corte; área bajo la curva: 0,8130

El termómetro (primera parte del instrumento) presentó un puntaje de malestar emocional con una mediana de 3 (IC_95%_ 3,39-4; rango intercuartílico=5). El área bajo la curva fue de 0,81 (IC_95%_ 0,77-0,86; DE=0,023) (figura 1). El punto de corte óptimo estimado fue de 3,5, pero al ser una escala de 0 a 10, sin puntos intermedios, se consideró que el punto de corte estimado podía aproximarse a 4, lo que coincide con el punto de corte recomendado por los autores del instrumento. Con este punto de corte igual o mayor de 4, el valor predictivo positivo fue de 58 % (IC_95%_ 53-63 %) y, el valor predictivo negativo, de 87 % (IC_95%_ 83-90 %). En el cuadro 3 se presentan los valores de la sensibilidad, la especificidad, y los valores predictivos positivos y negativos en cada punto de corte estimado.

Fuente: elaboración propia, programa Stata 13

**Cuadro 3:** Sensibilidad, especificidad, valor predictivo positivo (VPP) y valor predictivo negativo (VPN) en cada punto de corte de la escala

Puntos de corte	Sensibilidad	IC_95%_		Especificidad	IC_95%_		VPP	IC_95%_		VPN	IC_95%_	
1	0,97	0,95	- 0,99	0,25	0,21 -	0,30	0,40	0,35	- 0,45	0,95	0,92	- 0,97
2	0,94	0,91	- 0,96	0,42	0,37 -	0,47	0,47	0,42	- 0,52	0,93	0,90	- 0,95
3	0,86	0,82	- 0,90	0,58	0,53 -	0,63	0,52	0,46	- 0,57	0,89	0,85	- 0,92
4	0,81	0,76	- 0,85	0,69	0,64 -	0,74	0,58	0,53	- 0,63	0,87	0,83	- 0,90
5	0,74	0,70	- 0,79	0,75	0,70 -	0,79	0,61	0,56	- 0,66	0,84	0,80	- 0,88
6	0,54	0,49	- 0,59	0,87	0,83 -	0,90	0,68	0,63	- 0,73	0,78	0,73	- 0,82
7	0,43	0,38	- 0,48	0,92	0,89 -	0,95	0,73	0,68	- 0,77	0,75	0,70	- 0,79
8	0,30	0,25	- 0,35	0,96	0,93 -	0,98	0,78	0,73	- 0,82	0,72	0,67	- 0,77
9	0,13	0,1 -	0,17	0,98	0,96 -	0,99	0,75	0,70	0,79	0,68	0,63	- 0,73
10	0,05	0,03	- 0,08	0,99	0,97	- 1	0,75	0,70	0,79	0,66	0,61	- 0,71

La prevalencia de malestar emocional con el instrumento y un punto de corte óptimo igual o mayor de 4 fue de 48,4 % (n=166), en tanto que la prevalencia de malestar emocional, moderado a grave según el criterio clínico basado en la entrevista semiestructurada, fue de 34,7 % (n=119).

Los problemas más frecuentes detectados en el instrumento, asociados con un puntaje igual o mayor de 4 según el dominio, fueron los problemas prácticos: seguros/finanzas, 74 pacientes (p=0,016); los problemas de salud en la familia, 53 pacientes (p=0,117); los problemas emocionales: preocupación, 65 pacientes (p<0,001); los problemas religiosos (un único ítem), seis pacientes (p<0,001); los problemas físicos: fatiga/cansancio, 78 pacientes (p<0,001) (cuadro 4).

**Cuadro 4:** Resultados según el punto de corte del termómetro de malestar emocional

Lista de problemas	Termómetro				
	≤ 3		≥ 4		
Problemas prácticos	n	%	n	%	p
Cuidado de los niños	19	83	4	17	0,001
Cuidado del hogar	51	73	19	27	0,00
Seguros o finanzas	91	55	74	45	0,016
Transporte	86	59	59	41	0,001
Trabajo o estudio	72	65	39	35	0,00
Decisiones acerca de los tratamientos médicos	35	74	12	26	0,00
Problemas familiares					
Interacción con los niños	15	56	12	44	0,438
Interacción con la pareja	32	71	12	29	0,001
Capacidad para tener niños	11	58	8	42	0,394
Problemas de salud en la familia	63	54	53	46	0,117
Problemas emocionales					
Depresión	101	89	13	11	0,00
Miedos	86	75	28	25	0,00
Nerviosismo	120	70	52	30	0,00
Tristeza	132	72	52	28	0,00
Preocupación	133	67	65	33	0,00
Pérdida de interés en actividades habituales	70	69	31	31	0,00
Preocupación a nivel espiritual o religioso	21	78	6	22	0,01
Problemas físicos					
Apariencia	76	72	30	28	0,00
Bañarse o vestirse	45	66	23	34	0,001
Respiración	63	72	24	28	0,00
Cambios en la orina	53	60	36	40	0,014
Estreñimiento	76	65	41	35	0,00
Diarrea	28	53	25	47	0,482
Alimentación	78	60	51	40	0,001
Fatiga o cansancio	122	61	78	39	0,00
Sensación de estar hinchado	69	66	36	34	0,00
Fiebre	20	53	18	47	0,566
Capacidad para moverse	54	74	19	26	0,00
Indigestión	45	60	30	40	0,025
Memoria o concentración	82	64	47	36	0,00
Úlceras en la boca	22	58	16	42	0,214
Náuseas	80	61	52	39	0,00
Nariz seca/congestionada	72	57	55	43	0,018
Dolor	93	62	56	38	0,00
Vida sexual	17	63	10	37	0,115
Piel seca o picazón	97	58	71	42	0,001
Sueño	109	62	66	38	0,00
Uso de sustancias psicoactivas	2	67	1	33	0,525
Hormigueo en manos o pies	80	60	53	40	0,001
Otros problemas	16	49	17	52	0,991

En cuanto a los problemas más frecuentes que requirieron remisión en cada dominio de la entrevista semiestructurada, se encontraron los problemas prácticos, pues el 79 % de los pacientes presentaba dificultades para continuar con su trabajo y requería remisión; los problemas familiares, ya que el 54 % tenía preocupación porque sus familiares también presentaban problemas de salud; los problemas emocionales, pues el 92 % de los entrevistados había sentido tristeza durante la semana anterior a la entrevista, y el 87 % se sentía deprimido y nervioso, por lo que se les dio orden de remisión; en cuanto a los problemas religiosos, solo el 13 % refirió sentir algún problema en este sentido; en relación con los problemas físicos, el 81 % había sentido un dolor limitante durante la semana anterior y el 77 % se había sentido fatigado o cansado.

Tanto en la entrevista como con el instrumento, se detectaron 97 (28,3 %) pacientes con malestar emocional moderado a grave y en 155 (45,2 %) se descartó la presencia de malestar emocional que requiriera intervención. El porcentaje de acuerdo entre el resultado de la entrevista y el instrumento fue de 73 % (p<0,001), con un kappa de 0,64, es decir, un grado de acuerdo sustancial al ser mayor de 0,61 según Landis, *et al.*^28^.

## Discusión

Se evaluaron las características operativas de la escala *Distress Management,* en una muestra de pacientes colombianos con cáncer atendidos en el Instituto Nacional de Cancerología, centro de referencia de todo el país para el tratamiento de cáncer, por lo que se la consideró representativa de Colombia, ya que ahí se atienden pacientes de todas las edades, con todo tipo de cáncer y de todos los estratos socioeconómicos, aunque especialmente de estratos bajos pertenecientes al régimen de salud subsidiado.

El *Distress Management* de la NCCN se actualiza todos los años desde su creación en 1998 ^4^ y ha sido validado en diversos países. Al ser un instrumento en idioma inglés, se requiere su traducción, adaptación transcultural y validación en poblaciones diferentes a la original ^3^^,^^8^^,^^29^^-^^31^. Existe una versión en español de la NCCN, pero esta se hizo en el 2013 ^25^ y algunos ítems cambiaron en los años siguientes, por lo que la validación en la población colombiana atendida en el Instituto Nacional de Cancerología se hizo con la versión 2.2018 del original. Aunque ya se publicó la versión 2020, esta no incluye ningún cambio con respecto a la versión 2.2018 empleada en el estudio.

Los autores recomiendan un punto de corte de 4 para detectar el malestar emocional de moderado a grave que requiere intervención, pero el punto de corte varía según la población y el tipo de cáncer entre 3 y 5 en algunos países ^23^^,^^29^^,^^32^^,^^33^. En el presente estudio, se tomó un punto de corte de 4, el cual ofrece valores de sensibilidad y especificidad adecuados. En España, China, Turquía, Corea, Portugal, Italia, Reino Unido, Suecia, Irlanda y México, se ha conservado el punto de corte de 4, con un área bajo la curva de 0,66 a 0,83; en Dinamarca y Francia, el punto de corte es de 3, y no hay reporte del área bajo la curva; y en Holanda, Bélgica, Alemania e Indonesia, es de 5, con un área bajo la curva de 0,71 a 0,81 ^2^^,^^27^^,^^29^^,^^31^^,^^32^^,^^34^.

En este estudio, con el instrumento se detectó una prevalencia de malestar emocional de moderado a grave con necesidad de intervención del 34,7 %, cifra que concuerda con lo reportado en otros estudios, la cual oscila entre el 20 y el 50 % ^3^^,^^6^^,^^7^ y afecta todos los dominios evaluados, con excepción del religioso.

Estos hallazgos coincidieron con lo hallado en la entrevista semiestructurada, aunque con algunas variaciones: los problemas relacionados con los seguros o las finanzas no se consideraron por sí solos como un problema de malestar emocional que requiriera intervención, pero sí se les recomendó a los pacientes acudir a la sección de trabajo social del Instituto, en donde se ayuda a los pacientes con alojamiento, alimentación y algunos recursos económicos.

Según el índice kappa, la escala presentó un grado de acuerdo considerable ^28^ con la entrevista semiestructurada, considerada como el estándar de referencia a pesar de no disponer de una herramienta que evaluara integralmente el malestar emocional.

Actualmente, hay herramientas validadas que detectan específicamente la ansiedad, la depresión o la calidad de vida ^16^^,^^17^^,^^19^^,^^33^, y otras que evalúan más dominios relacionados con el malestar emocional, pero todas ellas requieren más tiempo para su diligenciamiento y no son de uso rutinario dado el tiempo limitado de atención de cada paciente ^21^.

En otros estudios de validación del termómetro de malestar emocional, este instrumento se comparó con la escala de Beck o la escala de HADS ^21^^,^^24^^,^^30^^,^^31^; no obstante, estas dos herramientas son específicas para evaluar la ansiedad y la depresión, y no todos los dominios relacionados con el malestar emocional incluidos en el termómetro. Se resalta que las entrevistas para evaluar el malestar emocional están a cargo de los servicios de psicología y psiquiatría de la institución, aunque estas no se hacen rutinariamente a todos los pacientes oncológicos y se limitan a aquellos que son remitidos por el servicio oncológico cuando presentan algún problema de salud mental. Por ello, hay un subregistro de los pacientes que requieren intervención, pues solo se los reporta cuando ellos mismos lo solicitan o el profesional de la salud a su cargo lo hace. Por tanto, se requieren herramientas de tamizaje eficaces y fáciles de usar, dada la prevalencia de trastornos relacionados con el malestar emocional, y la importancia de detectarlos y enfrentarlos oportunamente para, así, contribuir a un mejor resultado del tratamiento oncológico.

El instrumento tiene diferentes ventajas frente a otros: es sencillo y fácil de entender, pues consta de una lista de problemas y dos opciones de respuesta (sí o no). En la prueba piloto del estudio se evidenció que con una breve explicación antes de su diligenciamiento mejoraba la comprensión y el tiempo empleado por los pacientes para responder ^26^.

También, puede ayudar a detectar el malestar emocional reciente en los pacientes y, así, manejar tempranamente las alteraciones emocionales y mentales que lo requieran, ya que este puede variar en los diferentes estadios de la enfermedad y las etapas del tratamiento, sobre todo si se tiene en cuenta que el intervalo entre los controles médicos a veces es mayor de un mes.

Otra ventaja de este instrumento es que, en la lista de problemas, se incluyen la mayoría de los factores asociados con el malestar emocional, lo que proporciona información útil para orientar el tratamiento, decidir la remisión a psicología, psiquiatría o ambos, o verificar quién requiere asistencia de trabajo social.

Las limitaciones del estudio incluyen el poco tiempo de seguimiento a los pacientes que diligenciaron el instrumento, para evaluar los cambios a lo largo del tiempo y la capacidad de la herramienta para detectarlos.

El instrumento permitió detectar el malestar emocional moderado a grave que requiere intervención. En este estudio, se evaluaron sus características operativas en pacientes con cáncer en Colombia con el punto de corte igual o mayor de 4 tal como en la versión original, es decir, los pacientes con un resultado igual o mayor de 4 requerirían la remisión al grupo de salud mental (psicología, psiquiatría o ambos). Se necesitan estudios adicionales en otras instituciones para evaluar si varía la prevalencia de malestar emocional que requiere intervención.
